# Vessel Wall MRI: clinical implementation in cerebrovascular disorders—technical aspects

**DOI:** 10.1007/s11547-022-01484-7

**Published:** 2022-04-02

**Authors:** Luigi Cirillo, Arianna Rustici, Francesco Toni, Matteo Zoli, Fiorina Bartiromo, Laura Ludovica Gramegna, Domenico Cicala, Caterina Tonon, Ferdinando Caranci, Raffaele Lodi

**Affiliations:** 1 Department of Biomedical and NeuroMotor Sciences (DIBINEM), University of Bologna, Bologna, Italy; 2 UOC di Neuroradiologia, IRCCS Istituto delle Scienze Neurologiche di Bologna, Bologna, Italy; 3 Programma Neuroimmagini Funzionali e Molecolari, IRCCS Istituto delle Scienze Neurologiche di Bologna, Bologna, Italy; 4 Department of Experimental, Diagnostic and Specialty Medicine (DIMES), University of Bologna, Bologna, Italy; 5 Programma Neurochirurgia Ipofisi, IRCCS Istituto delle Scienze Neurologiche di Bologna, Bologna, Italy; 6 Division of Neuroradiology, Department of Neurosciences, Santobono-Pausilipon Children’s Hospital, Naples, Italy; 7 Radiology Department, Università Degli Studi Della Campania Luigi Vanvitelli, Neuroradiology Service CTO, Naples, Italy

**Keywords:** Vessel Wall Imagin, 3 Tesla MRI, Vasculitis, RCVS, Intracranial aneurysm

## Abstract

Vessel Wall MRI (VW-MRI) is an emerging MR sequence used for diagnosis, characterization, and treatment planning of cerebrovascular diseases. Although VW-MRI is not yet routinely used, most papers have emphasized its role in several aspects of the management of cerebrovascular diseases. Nowadays, no VW-MRI sequence optimized for the intracranial imaging is commercially available, thus the Spin Echo sequences are the more effective sequences for this purpose. Moreover, as one of the principal technical requirements for intracranial VW-MR imaging is to achieve both the suppression of blood in vessel lumen and of the outer cerebrospinal fluid, different suppression techniques have been developed. This short report provides the technical parameters of our VW-MR sequence developed over 3-years’ experience.

## Introduction

The diagnosis of cerebrovascular diseases is traditionally based on conventional imaging modalities, such as CT angiography (CTA), MR angiography (MRA), and Digital Subtraction Angiography (DSA). These techniques can detect only luminal abnormalities, and thus recently have been renamed “lumen-techniques”. Although the presence of luminal abnormalities is a sufficient criterion for the diagnosis for many cerebrovascular diseases, the lumen-techniques result inadequate in the diagnosis of pathologies predominantly involving the vessel walls [1], as for example Central Nervous System (CNS) vasculitis and Reversible Cerebral Vasoconstriction Syndrome (RCVS). Moreover, lumen techniques can sometimes fail to assess the bleeding risk of cerebrovascular diseases and define which lesions can be safely managed with conservative therapy versus those at high risk of rupture or growth necessitating treatments [2].

To date, MR is the only clinical available imaging modality which allow to image the brain vessels beyond the “lumen-techniques”, depicting both the lumen and the vessel walls [3, 4] with high sensitivity and low invasiveness [5, 6]. In fact, over the standard MRA sequences, some MR sequences named Vessel Wall (VW) Imaging have been introduced into the clinical practice [7]. These MR sequences allow to depict with High Resolution (HR) the vessel walls, becoming emerging techniques to evaluate cerebrovascular diseases.

Although MRI allows good contrast resolution of soft tissues [3, 4], a sub-millimetric spatial resolution is required to depict the vessel wall, because of the tortuosity, the small diameter, and the complex anatomy of the intracranial vessels. Morphological MR sequences do not allow HR visualization of the intracranial vessels and of their walls [8], as the wall thickness of a cerebral artery ranges between 0.2 and 0.7 mm [9, 10]. However, due to recent technological advancement, MRI has achieved a non-invasive sub-millimetric definition for soft tissues [3, 4].

Despite Vessel Wall MRI (VW MRI) sequences being widely reported as effective and efficient [9], there are no commercially available sequences optimized for the intracranial imaging. In fact, VW MRI needs a very complex signal to be generated, because there is the needing to suppress both the signal from blood inside the lumen and from the outer cerebrospinal fluid (CSF).

The aim of this short report is to describe the technical aspects of our VW MRI sequence, allowing other institution to develop their own sequence for the diagnosis and follow-up of different cerebrovascular pathologies.

## Materials and methods

The study was approved by the local institutional review board and written informed consent was obtained from all subjects.

The Vessel Wall MR sequence was performed on a 3.0 Tesla (T) MRI scanner (MAGNETOM Skyra, Siemens Healthcare, Erlangen, Germany) equipped with a 64-channel Head and Neck coil (Siemens Healthcare, Erlangen, Germany).

In our protocol, we always acquire the VW sequence after MRA (usually a Time-Of-Flight—TOF), to be able to center the VW sequence on the polygon of Willis.

Whenever possible we acquire the VW sequence both pre-and post-Gadolinium injection. We typically use gadoteridol (ProHance®; Bracco Diagnostic Inc.) as Gadolinium-based contrast agent, administering the standard dose recommended by the manufacturer based on bodyweight.

### Vessel Wall MRI technique

The prototype of our sequence is a T1-weighted 3D SPACE sequence, where specific parameters modifications have been optimized to achieve sufficient Signal-to-Noise Ratio (SNR) and Contrast-to-Noise Ratio (CNR) for Vessel Wall Imaging (VWI).

Moreover, we have set parameters to reach isotropic 3D imaging, good CFS, and blood suppressions, and to reduce scanning time (to avoid patient’ motion-induced artifacts) [11, 12] for a total scanning time of 7 min 10 s.

Our VW MRI sequence is a 3D multi-slab acquisition (80 slice for slab, each with 0.60 mm slice thickness) acquired in coronal plane, with a rectangular FOV (FOV read = 160 mm; FOV phase = 82.8%) (Table 1). To reduce the slab boundary artifact, we use oversampling method in the slice direction (slice oversampling = 10.0%), whereas to avoid the wrap-around artifact we use the phase oversampling technique (phase oversampling = 20%; phase resolution = 100%).

**Table 1:** Vessel Wall MRI sequence parameters at our Institution

Acquisition time	7:10 min
Acquisition orientation	Coronal
Type	3D
Slice for slab	80
Slice oversampling	10.0%
Slice thickness	0.60 mm
FoV read (mm)	160 mm
FoV phase (%)	82.8%
Phase Oversampling	20%
Phase resolution	100%
Voxel size	0.3 × 0.3 × 0.6
TR (ms)	1000 ms
TE (ms)	38 ms
ETL	211 ms
Flip angle (°)	T1 variable
Bandwidth (Hz/pixel)	514 Hz/Px
K-space filling	Interpolation with Zero filling
PAT mode	GRAPPA
Accel. factor PE	2
Ref. lines PE	24
Fat suppression	None
Dark blood	Off

As in Spin Echo (SE) MR sequences it is possible to adjust the TR (Time Repetition) and TE (Time Echo) to specific needing, we have set the TR at 1000 ms and the TE at 38 ms (Table 1), to achieve a CSF darkening effect by tailoring image contrast to T1/PD weighting (Table 2).

**Table 2:** Signal intensity, advantage, and disadvantage of each MR weighting

	T1-w	PD-w	T2-w
CSF	Dark	Light gray	Bright
Blood inside vessels	Bright	Dark	Dark
Advantages	High anatomical detail High contrast to Gd Relatively short lead times	High SNR High anatomical detail	High tissue contrast
Disadvantages	Low tissue contrast	Reduced contrast enhancement Reduced suppression of CSF	Mid-level anatomical detail Long time standard sequences

The Black-Blood (BB) effect is achieved by the intravoxel dephasing of moving blood spins within a long Echo Train Length (ETL = 211 ms) and variable refocusing Flip Angle (FA) were used to compensate for the signal decay inherent in the long ETL.

The difference in the precession frequencies of the spins inside the voxels at the extremities of the FOV is set at 514 Hz/Px (Bandwidth).

To reduce scanning time a Parallel imaging Acquisition Technique (iPAT) named GRAPPA (Generalized Autocalibrating Partially Parallel Acquisitions) is used, with an acceleration factor (*R*) of 2 and 24 references lines in the phase-encoding direction, to compensate the undersampling of k-space (Table 1).

With those parameters, there is no needing for an inferior outer volume suppression pulse to limit the inflow effects of blood, as well no fat-saturation pulse (Tables 1, 2).

## Discussion

Intracranial VW MRI is a complex technique requiring elevated spatial and contrast resolution, and the ability to detect contrast enhancement after the administration of contrast medium.

The correct visualization of the intracranial vessel walls relies on the suppression of the outer and the inner structures. Thus, both the signal of the blood inside the lumen and of the outer CSF must be suppressed. Although both structures are fluids, different suppression techniques are required because of their different properties. Lots of different fluid-suppression techniques have been developed by MR vendors, each presenting some limitations [13, 14].

Due to the difficulty to generate signal while suppressing others, and to the heterogeneity of scanners and coils, there are no commercially available sequences optimized for the intracranial imaging provided by MR vendors [10]. In this short report, we provide the technical parameters of our sequence for VWI, achieved in over 3-years’ efforts.

The basis of our VW MR sequence is a 3D SPACE sequence, thus a SE sequence. As all the SE are pulsed sequences, they allow to obtain different weighting basing on pre-defined timing parameters, as for example the TR and the TE. We decided to maintain the contrast of our sequence intermediate between the T1 and PD weighting because the T1-weighted sequences have the advantage of a more evident enhancement after the administration of contrast medium, whereas PD-weighted provides a higher SNR [10] (Table 2). Moreover, an intermediate T1/PD weighting achieves the required CSF suppression, due to its long T1 relaxation time (Table 2).

To achieve flowing-blood suppression lots of different and complex techniques have been developed, each presenting some limitations [13, 14]. The most used suppression techniques are the so-called “Black-Blood” (BB) [13] and can be broadly classified as either flow-dependent or flow-independent [14]. Although blood-suppression techniques are not the subject of this paper, it must be considered that the BB techniques have several limitations and they could lead to common artifacts [14], among which the most common is the presence of a residual blood signal due to insufficient blood suppression that can mimic or obscure vascular pathologies. This could happen in case of stagnant, slow, or retrograde blood flows, and typically with flow-dependent techniques.

Regardless of the suppression technique used, it must be considered that VW MRI has pitfalls that should be known [14], as for example the impossibility to assess the presence of wall enhancement in the cavernous segment of the internal carotid artery, because after contrast agent administration there is a diffuse enhancement inside the cavernous sinus [14].

Beyond contrast resolution, the scanning time is important too, because in clinical practice the use of sequences with long acquisition times may cause motion-induced artifacts. Thus, even if the MRI is an inherently slow imaging modality, it is necessary to reduce the scanning time. For that purpose, different Parallel Imaging (PI) techniques have been provided by different MR vendors.

**Fig. 1:**
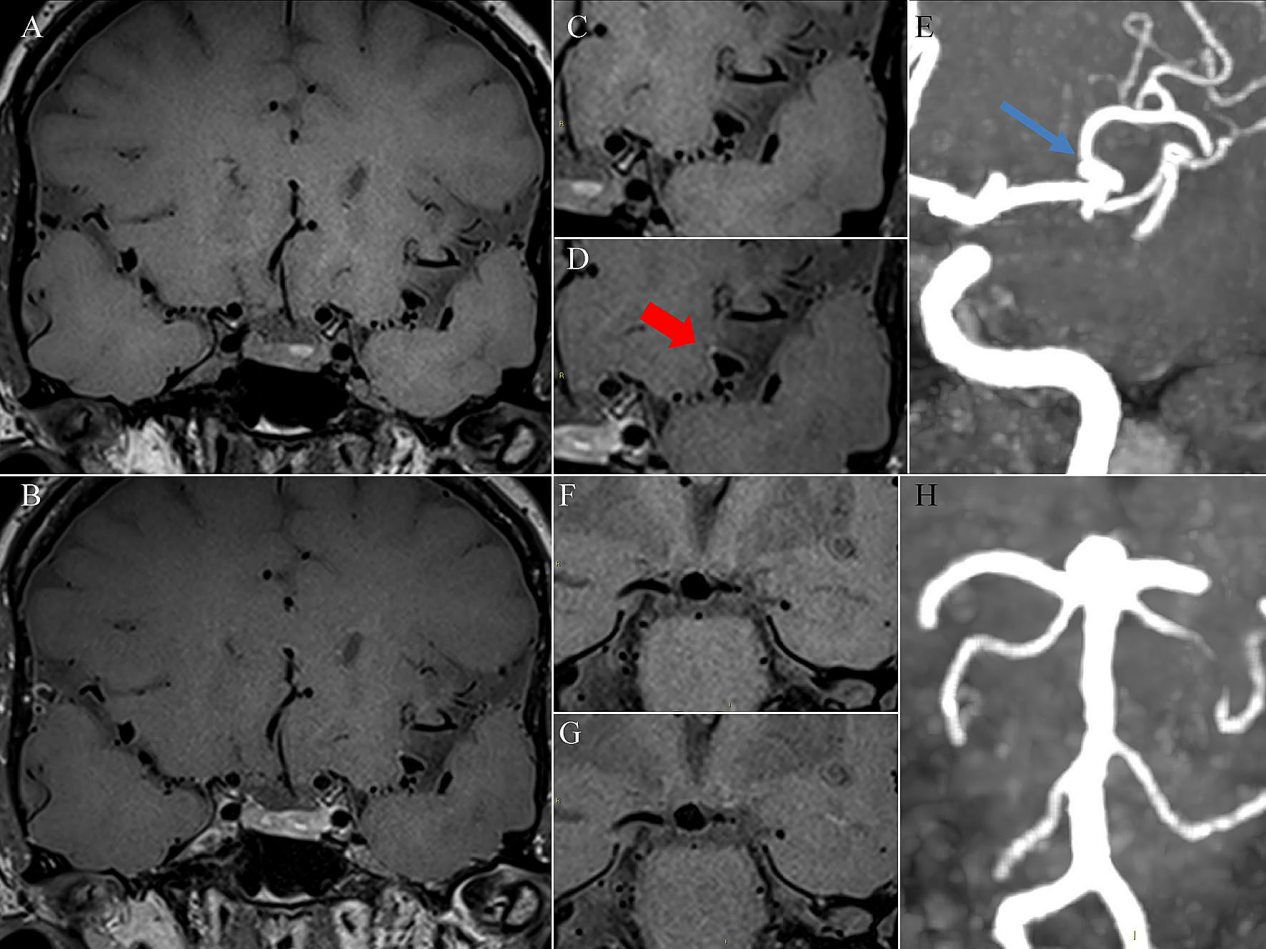
Aneurysms. A 64-year-old woman underwent brain MRI examination for the presence of incidentally discovering of multiple aneurysms, the largest located at left middle cerebral artery bifurcation (**E**) and at the basilar apex (**H**). Vessel Wall MRI sequences before (**A**) and after (**B**) the contrast medium administration, demonstrate the presence of a focal parietal enhancement after contrast medium of the left middle cerebral artery aneurysm (**C**, **D** red arrow, and **E**), while the basilar apex aneurysms did not show any parietal enhancement after the contrast medium administration (**F**, **G**, **H**). Despite the left bifurcation, MCA aneurysm did not fulfill the dimension criteria for treatment, the presence of wall enhancement let us decide to treat the aneurysm

**Fig. 2:**
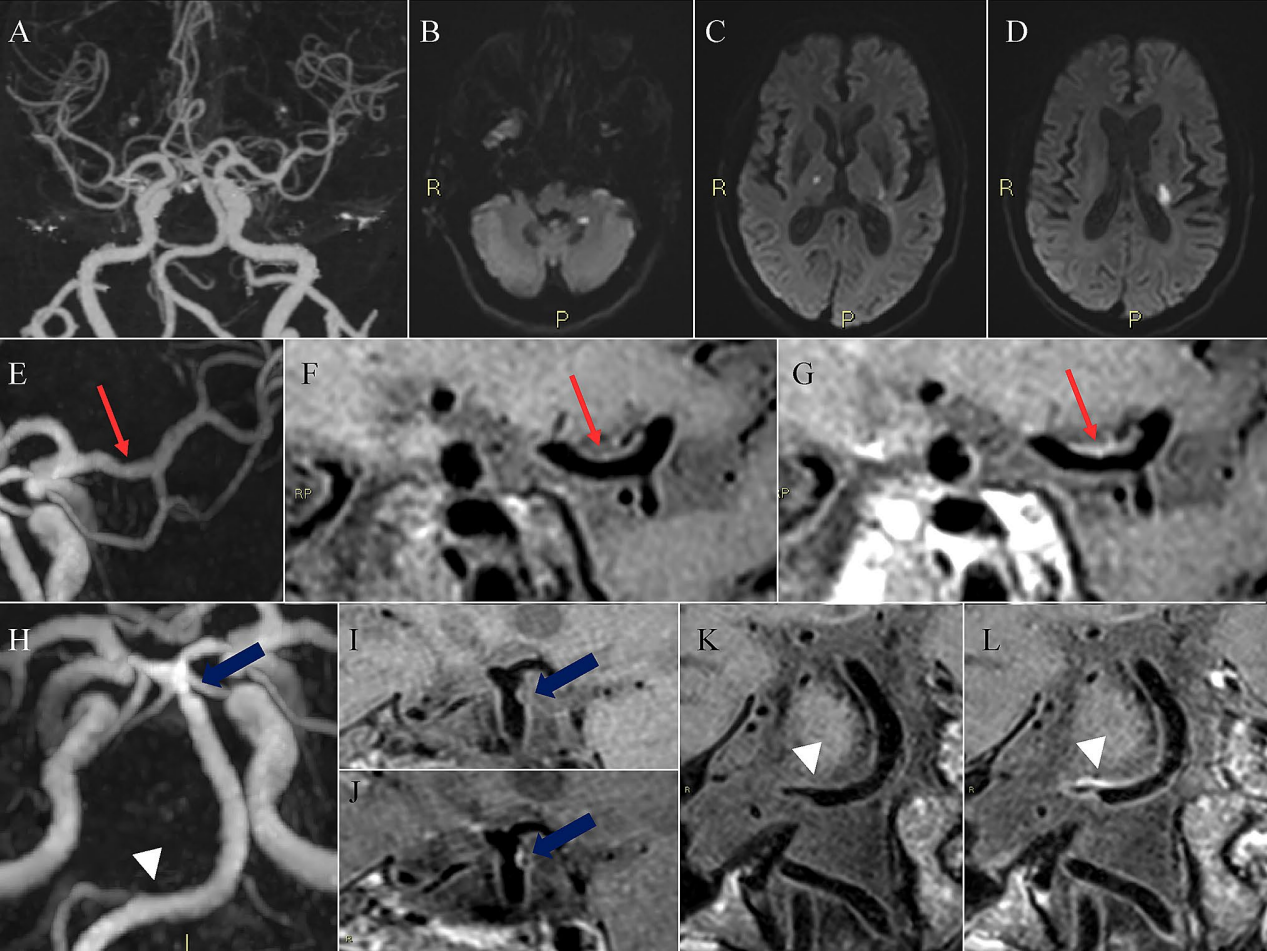
CNS vasculitis. A 58-year-old man with dizziness, vomiting, and speech disturbance underwent MRI study demonstrating in the TOF sequence, the presence of multiple caliber alterations in the intracranial arterial circulation (**A**), associated with recent ischemic lesions in the brain tissue (**B**–**D**) both in the anterior and in the posterior circulation. The caliber alterations detected in the TOF sequence demonstrated the presence of wall enhancement after the administration of contrast medium (**G**, **J**, **l**), particularly in the left M1 segment of the middle cerebral artery (**E**, **F**, **G**, red arrows), at the basilar artery apex (**H**, **I**, **J** blue arrows) and in the proximal portion of the basilar artery at the level of the right anterior inferior cerebellar artery (**H**, **K**, **L** white arrowheads). The laboratory test demonstrated the positivity for a T. pallidum infection and thus the final diagnosis is a luetic CNS vasculitis

**Fig. 3:**
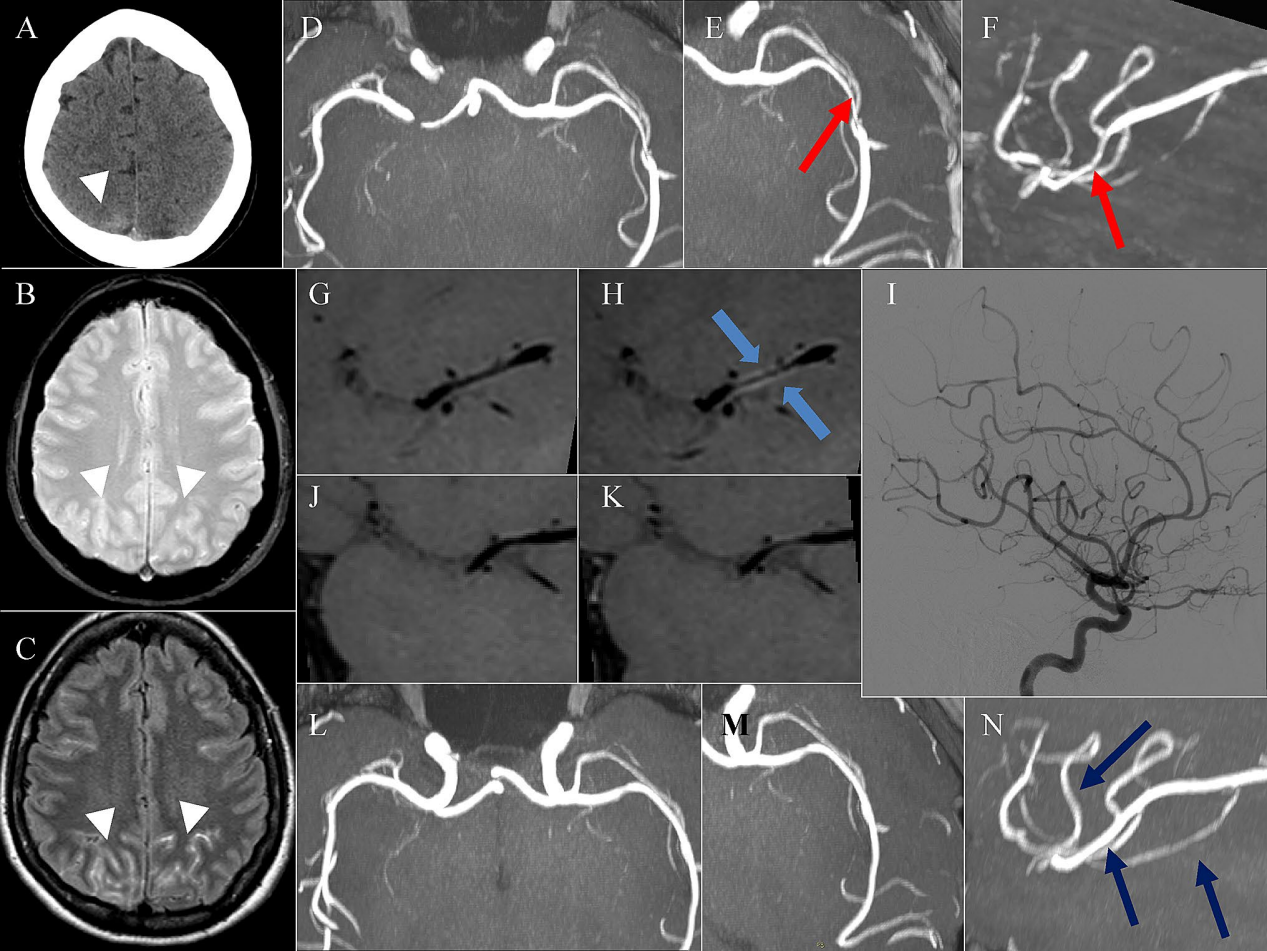
RCVS. A 56-year-old woman presented with an abrupt onset of headache and a CT scan demonstrated the presence of a small amount of subarachnoid hemorrhage in the right posterior parietal sulci (**A**). Nonvascular lesions responsible for the SAH were detected and thus the patient underwent brain MRI after few days, confirming the presence of a small amount of subarachnoid hemorrhage in the right posterior parietal sulci for the presence of hyperintensity in T2* and FLAIR sequences (**B**, **C** white arrowheads). The TOF sequence highlight the presence of caliber alterations in the left M2 segment (**D**–**F**, red arrows), and the Vessel Wall MRI study before (**G**) and after (**H**) the administration of contrast material demonstrated the presence of a slight circumferential enhancement in correspondence of the left M2 segment stenosis (blue arrows). Few days after the patient underwent a DSA angiography (**I**) that demonstrated the presence of a more diffuse caliber alterations in the intracranial arterial vasculature. The six months MRI follow-up demonstrates the complete resolution of the enhancement at Vessel Wall study before (**J**) and after (**K**) the contrast administration, as well as the caliber alterations (**L**–**N** blue arrows). The reversibility of those findings was then suggestive for a Reversible Cerebral Vasoconstriction Syndrome (RCVS), although non-enhancing concentric wall thickenings are more common than enhancing ones

We have reported our experience in the set of a VW MRI sequence, a novel imaging tool that has been evolving in recent years for the diagnosis and treatment decision of different cerebrovascular pathologies, such as aneurysms [7, 11, 15] (Fig. 1), CNS vasculitis (Fig. 2), reversible cerebral vasoconstriction syndrome—RCVS (Fig. 3) [12], intracranial atherosclerosis, dissections, Moya-Moya and Moya-Moya like diseases [8–10]. Despite heterogeneity in VW sequences, a recently published meta-analysis in the use of VW MRI on intracranial aneurysms identify a positive predictive value of 14.4%, a negative predictive value of 96.0%, whit an overall accuracy of the test of 59.7% [16].

In our experience in over 200 VW MRI examinations with the aforementioned sequence, we have found VW MRI examinations useful in the diagnosis of more than 97.1% of cases (31.7% VW-MRI examinations were determined to be positive for vessel wall pathologies, while 65.4% were negative), and only in the 2.9% of cases, the VW MRI were undetermined for different causes. Thus, in our experience less than 3% of VW MRI studies are undetermined, due to artifacts related to patients’ movement or blood suppression artifacts.

Compared to other VW MR sequences reported in the literature, ours has the advantage of being as simple as possible, because no blood-suppression techniques are used, thus avoiding suppression-related artifacts.

## Abbreviations

CTA: CT angiography
MRA: MR angiography
DSA: Digital Subtraction Angiography
CNS: Central Nervous System
RCVS: Reversible Cerebral Vasoconstriction Syndrome
VW: Vessel Wall
HR: High Resolution
VW-MRI: Vessel Wall MRI
CSF: Cerebrospinal Fluid
TOF: Time-Of-Flight
SNR: Signal-to-noise ratio
CNR: Contrast-to-noise ratio
VWI: Vessel Wall Imaging
SE: Spin Echo
TR: Time Repetition
TE: Time Echo
BB: Black-Blood
ETL: Echo Train Length
FA: Flip Angle
iPAT: Parallel imaging Acquisition Technique
GRAPPA: Generalized Autocalibrating Partially Parallel Acquisitions
